# Preliminary Psychometric Properties of Panic Disorder Severity Scale—Self‐Report Version: Validity, Reliability, and the Cut‐Off Point in Persian Clinical Samples

**DOI:** 10.1002/brb3.71524

**Published:** 2026-06-08

**Authors:** Fatemeh Akhavan‐Abiri, Mohammad Reza Shaeiri, Hojjatollah Farahani, Arsia Taghva

**Affiliations:** ^1^ Department of Psychology Faculty of Humanities Shahed University Tehran Iran; ^2^ Department of Psychology Faculty of Humanities Tarbiat Modares University Tehran Iran; ^3^ Department of Psychiatry 505 Army Psychiatric Hospital, AJA University of Medical Sciences Tehran Iran

**Keywords:** cut‐off point, panic, PDSS‐SR, reliability, sensitivity, specificity, validity

## Abstract

**Introduction:**

Accurate assessment of panic disorder (PD) severity requires brief and psychometrically sound instruments. The Panic Disorder Severity Scale—Self‐Report (PDSS‐SR) is widely used for this purpose. The present study aimed to evaluate the validity, reliability, and optimal cut‐off point of the PDSS‐SR in an Iranian sample.

**Method:**

Following a descriptive design, the clinical group of the sample consisted of 45 clinical patients diagnosed with PD (75.6% female), with a mean age of 34.04 years (standard deviation [SD] = 12.17) in Tehran in 2023 and 2024 referred to psychotherapy clinics, along with 18 non‐PD individuals for the comparison group (83.3% female), with an average age of 31.17 (SD = 7.94). Data were analyzed using confirmatory factor analysis, correlation matrices, reliability coefficients, receiver operating characteristic (ROC) curves, sensitivity, specificity, and the estimation of cut‐off points.

**Results:**

The construct validity of the PDSS‐SR was confirmed through CFA, with adequate goodness‐of‐fit for the PDSS‐SR single‐factor structure. By calculating the correlation of PDSS‐SR scores with scores on anxiety‐related scales, convergent validity was obtained on average. The sensitivity and specificity obtained from the PDSS‐SR identified the optimal cut‐off point of 6.500 (area under curve [AUC] = 0.78), with sensitivity (90.70%) and specificity (61.11%) based on the Youden index. Reliability was obtained by calculating Cronbach's alpha, McDonald's omega, test–retest correlation coefficient, and intraclass correlation coefficient (ICC) as 0.864, 0.861, 0.716, and 0.864, respectively.

**Conclusion:**

This research found that the PDSS‐SR had sufficient reliability, moderate convergent validity, and good construct validity in Iranian samples, with an optimal cut‐off point of 6.50. Therefore, it can be applied in clinical settings.

## Graphical abstract

METHOD & PARTICIPANTS (Iranian Sample, N = 63)

Self‐Report Scale Administration

• Clinical Group (PD) (N = 45; 75.6% female)

• Comparison Group (non‐PD) (N = 18; 83.3% female)

Cronbach's alpha: 0.864

McDonald's omega: 0.861

Test‐Retest: 0.716

• Construct & Convergent Validity

Single‐Factor Model Confirmed

Convergent Validity Confirmed (Correlation with anxiety‐related scales)

DIAGNOSTIC PERFORMANCE & KEY FINDINGS

• DIAGNOSTIC PERFORMANCE | Optimal Cutoff Point

• ROC curve | Accuracy | 6.50

Diagnostic performance derived from ROC & Accuracy analysis

• Validated Persian PDSS‐SR version

• Confirmed robust single‐factor model

• Excellent reliability

• Powerful diagnostic discrimination tool

WILEY | Brain and Behavior

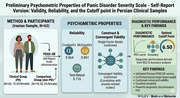

**Abbreviations**
ADIS‐5: Anxiety and Related Disorders Interview Schedule for DSM‐5
APA: American Psychiatric Association
ASI‐R: Anxiety Sensitivity Index—Revised
ATQ: Automatic Thoughts Questionnaire
AUC: Area Under Curve
BAI: Beck Anxiety Inventory
BDI‐II: Beck Depression Inventory—Second Edition
CEQ: Cognitive Errors Questionnaire
CFA: confirmatory factor analysis
CFI: Comparative Fit ndex
CI: Confidence Interval
df: Degree of Freedom
DSM: Diagnostic and Statistical Manual of Mental Disorders
FSAQ: Four System Anxiety Questionnaire
FSS‐III: Fear Survey Schedule—Third Version
GAD‐7: 7‐item Generalized Anxiety Disorder
GFI: Goodness of Fit Index
GSI: Global Severity Index
HSCL‐25: Hopkins Symptom Checklist‐25
ICC: Intraclass Correlation Coefficient
IFI: Incremental Fit Index
K–S: Kolmogorov–Smirnov
LR: Likelihood Ratio
NFI: Normed Fit Index
PD: Panic Disorder
PDSS: Panic Disorder Severity Scale
PDSS‐SR: Panic Disorder Severity Scale—Self‐Report
PPV: Positive Predictive Value
PROM: Patient‐Reported Outcome Measure
RMSEA: Root Mean Square Error of Approximation
ROC: Receiver Operating Characteristic
SCID‐5: Structured Clinical Interview for DSM‐5
SCID‐5‐RV: Structured Clinical Interview for DSM‐5 Research Version
SCL‐90‐R: Symptom Checklist‐90‐Revised
SD: Standard Deviation
SE: Standard Error
SERS: Self‐Esteem Rating Scale
SPIN: Social Phobia Inventory

## 1 Introduction

A panic attack is a sudden and intense surge of fear accompanied by physical and cognitive discomfort, typically reaching its peak within minutes (American Psychiatric Association [APA] 2022). The prevalence estimate for panic disorder (PD) is reported to be approximately 2% of the general population (Gorbis and Jajoo 2024). The global lifetime prevalence is estimated at 1.7%, with a projected lifetime risk of 2.7% according to the World Mental Health Surveys (APA 2022). However, recent literature emphasizes the high disease burden and the complexity of its clinical management in modern psychiatric practice. Despite its clinical significance, a substantial portion of affected individuals—approximately two‐thirds—do not receive adequate evidence‐based treatment. This discrepancy underscores the urgent need for more accessible and culturally adapted screening tools to facilitate earlier detection and bridge the gap between community prevalence and clinical care (Gorbis and Jajoo 2024).

Symptom monitoring improves treatment adjustment and outcomes (Fortney et al. 2017). Accordingly, accurate diagnosis and ongoing assessment of symptom severity play a crucial role in optimizing patient care and evaluating treatment effects. However, the routine integration of patient‐reported outcome measures (PROMs) into clinical practice requires instruments that are both psychometrically sound and feasible for regular use (Kwan et al. 2025). Research indicates that specific scales measuring symptoms are stronger predictors than general scales since they are more sensitive to change (Jensen‐Doss et al. 2018). Therefore, the use of diagnosis‐specific scales becomes more valuable in this context (Fogliati et al. 2016).

The Panic Disorder Severity Scale (PDSS) is a comprehensive assessment tool for evaluating PD symptoms utilizing an interview‐based format (Shear et al. 1997). Modeled after the Yale‐Brown Obsessive‐Compulsive Scale, the PDSS consists of seven items, each rated on a scale from 0 to 4, where 0 indicates no symptoms and higher ratings reflect increasing levels of symptom severity. The PDSS has been widely utilized in both clinical practice (Roberge et al. 2020) and research settings (Roberge et al. 2022) as a standardized instrument for assessing symptom severity and evaluating treatment response. Its symptom‐specific structure has supported its application in clinical trials and longitudinal outcome research (Roberge et al. 2018).

Despite its widespread application, the PDSS has a significant limitation: it must be administered by a trained clinician. This limitation prompted the development of a self‐report version of the instrument, known as the Panic Disorder Severity Scale—Self‐Report (PDSS‐SR; Houck et al. 2002). The PDSS‐SR assesses the same content as the PDSS and is scored on a 5‐point ordinal scale (0–4), with a total score ranging from 0 to 28. The primary modification compared to the PDSS is the time frame; while the PDSS assesses symptoms over the past month, the PDSS‐SR focuses on the past week. This change was implemented to minimize potential recall bias and to enable the instrument to monitor symptoms on a weekly basis (Houck et al. 2002).

The PDSS‐SR has been translated and validated in several cultural contexts. Recent research continues to support its cross‐cultural utility, confirming its reliability and measurement invariance across diverse clinical samples (Roberge et al. 2022).

Despite its widespread application across various cultures and in different clinical and research contexts, the psychometric properties of the PDSS‐SR have not yet been systematically examined in Iranian populations.

International guidelines emphasize that psychological instruments must undergo rigorous validation within the target linguistic and cultural context to ensure conceptual and structural equivalence (Dept 2024). Linguistic and cultural differences may influence response patterns and factor structure, potentially affecting the interpretation of scores if equivalence is not empirically examined (Tan et al. 2020). The PDSS‐SR was specifically selected for Persian adaptation over other available instruments due to its unique clinical and psychometric advantages. While various self‐report measures exist for assessing some panic features, few instruments offer the comprehensive assessment of PD severity provided by the PDSS‐SR (Roberge et al. 2022). Crucially, the scale is closely aligned with the DSM‐5 diagnostic criteria for PD, ensuring that it captures the full clinical spectrum of the condition. As the self‐report counterpart to the extensively utilized clinician‐administered PDSS, this version offers a brief yet valid approach for monitoring symptoms and treatment response in both research and busy clinical settings, making it an ideal choice for the Iranian clinical context where efficiency and diagnostic precision are paramount. Accordingly, since no validated Persian version of this scale—or any other panic‐specific instrument—was available, the current study undertook this transcultural adaptation to address a significant gap in the Iranian clinical literature. By strictly following a formal multistage protocol, we sought to ensure linguistic and conceptual integrity, providing an essential foundation for the tool's application in Iranian clinical samples. Therefore, the main focus of the current research is to evaluate the psychometric properties of the Persian version of the PDSS‐SR—establishing an essential foundation for its application—in Iranian clinical samples.

## 2 Methods

### 2.1 Participants

The study included two groups: a clinical group and a comparison group. The clinical group was recruited from outpatients diagnosed with PD who were referred to psychotherapy clinics in Tehran between 2023 and 2024 using purposive sampling (Tongco 2007). Participants in the clinical group were recruited from several clinical centers, including the Shahid Beheshti University Counseling Center, the Iran University of Medical Sciences Counseling Center, and the Psychiatric Outpatient Clinic of the 505 Army Hospital. The clinical group consisted of 45 outpatients who received a primary diagnosis of PD based on a structured diagnostic interview conducted by a trained clinical psychotherapist using the Structured Clinical Interview for DSM‐5 (SCID‐5).

Sample size determination was guided by recommendations for psychometric validation studies. In confirmatory factor analysis (CFA)‐based preliminary validation studies, a minimum ratio of 5 participants per item is commonly recommended (Bentler and Chou 1987), which for the 7‐item PDSS‐SR yields a minimum requirement of 35 participants. In addition, the achieved sample size (*n* = 45) meets the lower bound criteria for stable estimation in models with limited parameters and is consistent with previous validation studies of the PDSS‐SR conducted in clinical samples.

The inclusion criteria were as follows: (1) meeting DSM‐5‐TR criteria for PD; (2) age between 18 and 65 years; (3) fluency in speaking and understanding Persian; and (4) willingness to participate and provide informed consent. Exclusion criteria included (1) the presence of a primary psychiatric disorder other than PD that could compromise the validity of self‐reported panic symptoms; (2) current substance use disorder; (3) acute suicidal ideation; and (4) cognitive or neurological impairment interfering with participation.

In addition, a comparison group of 18 individuals drawn from the general population was recruited using purposive sampling through community‐based outreach (e.g., public advertisements and personal networks). In this group, participants were screened using the SCID‐5 to confirm the absence of a current diagnosis of PD. The inclusion criteria for the comparison group were as follows: (1) age between 18 and 65 years; (2) fluency in speaking and understanding Persian; and (3) willingness to participate and provide informed consent. Exclusion criteria included conditions that could interfere with the valid assessment of self‐reported symptoms: (1) current major psychiatric disorders, including psychotic and bipolar disorders; (2) current substance use disorder; and (3) cognitive or neurological impairment affecting comprehension.

The demographic characteristics of the participants are summarized in Table 1. To ensure the comparability of the two clinical and comparison groups, an independent *t*‐test and chi‐square analysis were performed. The results revealed no significant differences regarding age (*t*(61) = −0.93, *p* = 0.359) or gender distribution (*χ* ^2^(1) = 0.45, *p* = 0.502). These results confirm that the groups were well matched for subsequent analyses.

**TABLE 1:** Demographic characteristics.

Group	Variables		*N*	Precent	*M*	SD	Min	Max	Skew	Kurt
Clinical	Gender	Male	11	24.4%						
		Female	34	75.6%						
	Age		45	—	34.04	12.18	18	63	1.09	0.34
Comparison	Gender	Male	3	16.7%						
		Female	15	83.3%						
	Age		18	—	31.17	7.94	23	56	1.83	4.85

Abbreviations: Kurt, kurtosis; *M*, mean; Max, maximum; Min, minimum; SD, standard deviation; Skew, skewness.

### 2.2 Measures

The *Panic Disorder Severity Scale—Self‐Rating version (PDSS‐SR)* was introduced by Houck et al. (2002) and is based on the original PDSS developed by Shear et al. (1997). Similar to its parent version, the PDSS‐SR consists of seven items, each answered and scored on a 5‐point Likert scale ranging from 0 to 4. A composite score can be calculated by averaging the scores of all seven items. Roberge et al. (2022) investigated the psychometric characteristics of the PDSS‐SR in French samples and explored a one‐factor structure through exploratory factor analysis. The scale demonstrated good internal consistency (*α* = 0.91) and adequate convergent validity with clinician‐rated panic severity (Anxiety and Related Disorders Interview Schedule for DSM‐5 [ADIS‐5]) as well as related constructs such as anxiety sensitivity (Beck Anxiety Inventory [BAI]) in French samples. Due to the absence of a preexisting Persian version of the PDSS‐SR, a pioneering and preliminary transcultural adaptation was conducted for this study. To ensure linguistic and conceptual equivalence with the original English scale (Houck et al. 2002), we followed a formal translation and back‐translation protocol involving a panel of experts. This process yielded the initial version used for the current psychometric evaluation in the Iranian clinical context.

The *Structured Clinical Interview for DSM‐5 Disorders* (SCID‐5) is a semi‐structured interview designed for major diagnoses outlined in the DSM‐5 (First et al. 2016). The initial version of this interview was developed in 1983 based on the DSM‐III diagnoses. A recent revision began in 2012 and was published by First et al. (2016) after undergoing various updates to align with the newer editions of the DSM. The SCID‐5 has demonstrated strong psychometric properties in international studies. For example, Shankman et al. (2017) reported excellent internal consistency across diagnostic categories (*α* = 0.87–0.98) and acceptable sensitivity (0.38–0.92). Similarly, Osório et al. (2019) reported high diagnostic agreement between clinical interview and SCID‐5 diagnoses (73%–97%), with kappa coefficients, sensitivity, and specificity generally exceeding 0.70 across disorders. In Iranian samples, the SCID‐5 has also shown satisfactory psychometric properties. Mohammadkhani et al. (2020) reported strong internal consistency (*α* = 0.95–0.99), test–retest reliability (0.60–0.79), and kappa coefficients ranging from 0.57 to 0.72 for the research version (SCID‐5‐RV). In addition, Shabani et al. (2021) evaluated the clinical version (SCID‐5‐CV) and reported kappa values between 0.34 and 0.90, sensitivity ranging from 0.82 to 0.94, and specificity ranging from 0.78 to 0.98. In this study, the Persian version of the SCID‐5, which was previously validated by Mohammadkhani et al. (2020), was employed to determine the clinical diagnoses.

The *Anxiety Sensitivity Index—Revised* (ASI‐R) is a 36‐item self‐report instrument developed by Taylor and Cox (1998) in response to psychometric criticisms of the original 16‐item Anxiety Sensitivity Index (ASI; Reiss et al. 1986). Respondents answer items using a 5‐point Likert scale, ranging from 0 (*very little*) to 4 (*very much*). Zvolensky et al. (2003) identified a two‐factor structure comprising fear of physical sensations and social‐cognitive concerns. They reported good internal consistency, with Cronbach's alpha coefficients typically ranging from 0.83 to 0.93 across subscales. Moradi‐Manesh et al. (2007) evaluated the psychometric properties of the ASI‐R in Iranian samples and supported its reliability and validity. Their findings indicated satisfactory convergent validity with relevant dimensions of the SCL‐90‐R, with most correlations reaching statistical significance (*p* < 0.010). In addition, internal consistency, test–retest reliability, and split‐half reliability were reported as 0.93, 0.95, and 0.97, respectively, with Cronbach's alpha values for the four subscales ranging from 0.92 to 0.96. The Persian version of the ASI‐R validated by Moradi‐Manesh et al. (2007) was used in this study.

The *Four System Anxiety Questionnaire* (FSAQ) was introduced by Koksal and Power (1990) to assess the physical, cognitive, behavioral, and emotional components of anxiety. This questionnaire consists of 60 items, each answered with an item that carries a specific weight, which allows for the calculation of scores. The overall score for each component is determined by summing the scores of the four individual components (Koksal and Power 1990; Janda 2001; Maredpour et al. 2010). Koksal and Power (1990) reported satisfactory psychometric properties for the original version, including split‐half reliability coefficients of 0.68 to 0.82 for the subscales and 0.92 for the total score. Criterion validity was supported by significant group differences between anxious patients and nonclinical controls (*F*(1,270) = 171.98, *p* < 0.001). In Iranian samples, Maredpour et al. (2010) also reported acceptable internal consistency, with Cronbach's alpha coefficients of 0.64–0.78 for the subscales and 0.89 for the total score. Furthermore, significant differences between clinical and nonclinical groups (*p* < 0.010) supported the discriminant validity of the questionnaire in Iranian populations. The Persian version of the FSAQ, as validated by Maredpour et al. (2010), was used to measure anxiety symptoms.

*The third version of the Fear Survey Schedule* (FSS‐III) was introduced by Wolpe and Lang (1964) to assess fear‐inducing stimuli. The first version, known as FSS‐I, was developed by Lang and Lazovik (1963). Spinks (1980) reported an overall Cronbach's alpha coefficient of 0.95 for this scale. Bakhshi‐Pour et al. (2013) also reported strong psychometric properties for the FSS‐III in Iranian samples, including high internal consistency (*α* = 0.95) and test–retest reliability over a 1‐week interval (*r* = 0.94). In addition, exploratory factor analysis and CFA supported a six‐factor structure consistent with the original model, with CFA results indicating that the six‐factor solution provided the best fit compared with alternative models. The validated Persian translation of FSS‐III by Bakhshi‐Pour et al. (2013) was utilized in the current study.

The *Symptom Checklist‐90‐Revised* (SCL‐90‐R) was introduced by Derogatis et al. (1973) based on clinical experiences and psychometric analyses inspired by the Hopkins Symptom Checklist (HSCL‐25). The scoring scale for the items utilizes a 5‐point Likert scale to assess discomfort, ranging from 0 (*none*) to 4 (*extremely*). The SCL‐90‐R encompasses dimensions such as somatization, obsessive‐compulsion, interpersonal sensitivity, depression, anxiety, hostility, phobia, paranoid ideation, psychosis, and an additional dimension, in addition to a global severity index (GSI). Factor‐analytic evidence has generally supported the multidimensional structure of the instrument. For example, Tomioka et al. (2008) confirmed the adequacy of the model using CFA, supporting the theoretical structure of the nine symptom dimensions. In Iranian samples, Akhavan‐Abiri and Shairi (2020) reported satisfactory psychometric properties of the SCL‐90‐R. Convergent validity was supported through significant correlations with related measures (e.g., BAI, BDI‐II, GAD‐7). Internal consistency coefficients ranged from 0.68 to 0.88 across subscales, with a Cronbach's alpha of 0.97 for GSI. Test–retest reliability ranged from 0.66 to 0.85 across subscales and reached 0.84 for the GSI (*p* < 0.010). Both 9‐factor and alternative 10‐factor structural models were supported through CFAs in nonclinical Iranian samples. The Persian translation of the SCL‐90‐R validated by Mirzaei (1980) (as cited in Akhavan‐Abiri and Shairi 2020) was used in this study.

*Beck Depression Inventory—Second Edition* (BDI‐II) was first introduced by Beck et al. in 1961, revised in 1971, and published in 1978 (Beck et al. 1996). The BDI‐II consists of 21 items, each containing four statements, except for the items related to sleep and appetite, which include seven statements. Each statement is assigned a score based on its severity, with scores ranging from 0 to 3. The BDI‐II has demonstrated strong psychometric properties across diverse populations. In the original validation study, Beck et al. (1996) reported high internal consistency (*α* = 0.73–0.92, with an average of 0.86), with slightly higher reliability observed in clinical samples compared to nonclinical samples. In Iranian samples, Ghassemzadeh et al. (2005) reported good convergent validity, with significant correlations between the BDI‐II and the Automatic Thoughts Questionnaire (ATQ; *r* = 0.77). Factor‐analytic evidence supported a three‐factor structure, which showed better model fit compared with one‐ and two‐factor solutions (relative *χ* ^2^ = 1.80; goodness‐of‐fit index [GFI] = 0.81), supporting the construct validity of the Persian version. In the current study, we employed the Persian version of the BDI‐II, which had been previously cross‐culturally adapted and psychometrically validated by Ghassemzadeh et al. (2005) for use in Iranian clinical populations.

The *7‐item Generalized Anxiety Disorder* (GAD‐7) scale was developed by Spitzer et al. (2006) as a brief diagnostic tool to assess potential cases of generalized anxiety disorder and evaluate its severity. This scale consists of seven items, each scored from 0 (*never*) to 3 (*almost every day*). In the original validation study, Spitzer et al. (2006) reported good internal consistency (*α* = 0.92) and satisfactory test–retest reliability (*r* = 0.83). Convergent validity was supported through moderate to strong correlations with relevant measures, including the SF‐20 anxiety subscale (*r* = 0.39–0.91), the BAI (*r* = 0.72), and the anxiety subscale of the SCL‐90‐R (*r* = 0.70). Nainian et al. (2011) reported acceptable psychometric properties for the Persian version of the GAD‐7. Internal consistency was adequate (*α* = 0.85), while test–retest reliability was reported as 0.48 (*p* < 0.010). Evidence for convergent validity was demonstrated through significant correlations with the Spielberger State–Trait Anxiety Inventory (state: *r* = 0.71; trait: *r* = 0.52) and the anxiety subscale of the SCL‐90‐R (*r* = 0.63, *p* < 0.001), supporting the validity of the instrument in Iranian populations. The present study employed the Persian version of the GAD‐7 as adapted by Nainian et al. (2011) to ensure the cultural and linguistic relevance of the assessment.

The *Social Phobia Inventory* (SPIN) is a 17‐item self‐report instrument designed to assess social phobia through three subscales: avoidance, fear, and physiological discomfort. Respondents answer each item on a 5‐point Likert scale ranging from 0 (*not at all*) to 4 (*extremely*) (Connor et al. 2000). In the original validation study, Connor et al. (2000) reported strong psychometric properties for the SPIN, including high internal consistency (*α* = 0.94) for the total scale and acceptable reliability for its subscales (avoidance: *α* = 0.91; fear: *α* = 0.89; physiological discomfort: *α* = 0.80). Test–retest reliability was also satisfactory (*r* = 0.78, *p* < 0.001), supporting temporal stability. Hassanvand Amoozadeh et al. (2010) evaluated the psychometric properties of the SPIN in Iranian nonclinical populations. The scale demonstrated adequate reliability, with a split‐half coefficient of 0.84 and a Spearman–Brown coefficient of 0.91. Internal consistency for the subscales of fear, avoidance, and physiological discomfort was 0.74, 0.75, and 0.75, respectively. Evidence for convergent validity was supported by significant correlations with cognitive errors (Cognitive Errors Questionnaire [CEQ]; *r* = 0.35), self‐esteem (Self‐Esteem Rating Scale [SERS]; *r* = 0.58), and the phobic anxiety subscale of the SCL‐90‐R (*r* = 0.70), indicating acceptable construct validity in Iranian populations. The validated Persian version by Hassanvand Amoozadeh et al. (2010) was utilized in the current research to ensure the diagnostic accuracy of social anxiety assessments.

### 2.3 Procedure

First, the developers of the PDSS‐SR were contacted to obtain consent for conducting a study on Iranian samples. After receiving permission, the transcultural adaptation followed a multistage protocol. First, a clinical psychologist translated the original PDSS‐SR items from English to Persian, focusing on conceptual rather than literal equivalence. An English language expert then conducted a blind back‐translation of the Persian version back into English. After receiving no response from the PDSS‐SR developers to verify the retranslation after multiple attempts, the English language expert and the research team compared the retranslated version with the original PDSS‐SR to ensure semantic integrity. This comparison suggested two minor linguistic corrections: one involved correcting the verb tense, and the other involved changing a brief description of an option from an adverb to an adjective for better conceptual clarity in Persian. The final draft was reviewed to ensure that it was culturally appropriate for the Iranian clinical context. The validity and reliability of the PDSS‐SR were subsequently assessed in a clinical group of 45 individuals eligible for this part of the study, along with the accompanying tools, by the researcher. Participants completed the questionnaires individually in a quiet clinical setting under the supervision of a trained researcher.

Participants in the clinical group completed the full battery of assessments, including the PDSS‐SR and all convergent validity measures (ASI‐R, FSAQ, etc.). However, for the comparison group, data collection was limited to the PDSS‐SR and demographic information. This decision was made to reduce participant burden and because the primary objective for the comparison group was to evaluate the scale's discriminant capacity between clinical and nonclinical populations, rather than assessing convergent validity within a non‐PD‐diagnosed sample. Therefore, the convergent validity instruments (e.g., ASI‐R, FSAQ, etc.) were administered exclusively to the clinical group (*n* = 45).

After eligibility screening and diagnostic assessment with the SCID‐5, participants completed the questionnaires individually in a quiet setting under the supervision of a trained researcher. The administration took approximately 45 min for the clinical group and 5 min for the comparison group, as the latter only completed the PDSS‐SR to assess the scale's specificity. Clinical participants completed the anxiety‐related measures, while the comparison group completed the PDSS‐SR exclusively. All participants were instructed to respond based on their experiences during the past week, in accordance with the time frame of the PDSS‐SR.

#### 2.3.1 Ethical Considerations

The study protocol was approved by the Research Ethics Committee of Shahed University (Approval Code: IR.SHAHED.REC.1402.126). All procedures performed in this study involving human participants were in accordance with the ethical standards of the institutional research committee and with the 1964 Helsinki Declaration and its later amendments. Informed consent was obtained from all individual participants included in the study.

#### 2.3.2 Consent Inform

All participants provided written informed consent before participation after receiving a full explanation of the study procedures. Participation was voluntary, and confidentiality of the data was assured.

### 2.4 Data Analysis

The reliability of the PDSS‐SR was assessed by calculating Cronbach's alpha and the split‐half method in SPSS version 27. The factor structure of the PDSS‐SR was evaluated through CFA using AMOS version 18. To assess the model fit, several suitability indices were employed, including the chi‐square ratio (χ^2^/*df* < 3), normed fit index (NFI > 0.90), comparative fit index (CFI > 0.90), incremental fit index (IFI > 0.90), and root mean square error of approximation (RMSEA < 0.06). These indices and their corresponding threshold scores were selected based on established psychometric standards (Hu and Bentler 1999). Convergent validity was established by calculating Pearson correlations between the composite score of the PDSS‐SR and the subscales of various tools, including the ASI‐R, FSAQ, FSS‐III, SCL‐90‐R, BDI‐II, GAD‐7, and SPIN, also using SPSS version 27.

## 3 Results

### 3.1 Descriptive Findings

Before conducting the primary psychometric analyses, descriptive statistics—including means, standard deviations (SDs), minimum, maximum scores, skewness, and kurtosis—were calculated for the PDSS‐SR and all other clinical instruments. As specified in Section 2.3, the PDSS‐SR was completed by both groups, while the convergent validity measures were administered exclusively to the clinical group (*n* = 45). Tables 2 and 3 summarize these descriptive findings, providing an overview of the symptomatic profile of the participants. The distributional properties (skewness and kurtosis) were further examined to determine the suitability of subsequent parametric or nonparametric statistical tests.

**TABLE 2:** Descriptive statistics for the PDSS‐SR.

Variables	Group	*n*	*M*	SD	Min	Max	Skew	Kurt
PDSS‐SR raw score	Clinical	45	13.21	5.89	1.00	26.00	0.41	−0.41
	Comparison	18	6.44	5.82	0.00	17.00	0.50	−0.98
PDSS‐SR composite score	Clinical	45	1.89	0.84	0.14	3.71	0.41	−0.41
	Comparison	18	0.92	0.83	0.00	2.43	0.50	−0.98

Abbreviations: Kurt, kurtosis; *M*, mean; Max, maximum; Min, minimum; PDSS‐SR, Panic Disorder Severity Scale—Self Report; SD, standard deviation; Skew, skewness.

**TABLE 3:** Descriptive statistics for anxiety‐related measures in the clinical group (*n* = 45).

Variables		Statistics					
Scale	Subscales/total scores	*M*	SD	Min	Max	Skew	Kurt
ASI‐R	Cardiovascular–Digestive	17.77	9.97	0.00	40.00	0.38	−0.20
	Respiratory	14.84	6.99	1.00	28.00	0.07	−0.69
	Fear of Observable Publicly Anxiety Reactions	18.85	6.45	0.00	32.00	−0.14	0.60
	Cognitive Dyscontrol	8.96	5.58	0.00	20.00	0.34	−0.60
BDI‐II	Total Depression	28.43	14.07	0.00	60.00	0.27	0.12
FSAQ	Feeling	59.12	22.55	8.10	82.00	−0.83	−0.49
	Cognitive	60.62	22.18	6.90	82.40	−0.84	−0.44
	Behavioral	45.55	19.76	6.00	82.50	−0.19	−0.68
	Somatic	43.62	16.73	11.90	82.40	0.25	−0.64
	Total	209.00	67.57	66.00	329.30	−0.42	−0.57
GAD‐7	Total Generalized Anxiety	14.69	5.26	6.00	24.00	0.17	−0.99
SCL‐90‐R	Somatization	22.06	10.15	3.00	44.00	0.37	−0.33
	Obsessive–Compulsion	19.18	8.45	1.00	39.00	−0.09	−0.07
	Interpersonal Sensitivity	16.68	7.63	2.00	32.75	0.03	−0.56
	Depression	28.10	12.54	0.00	51.00	−0.29	−0.49
	Anxiety	19.71	9.32	1.00	38.00	−0.13	−0.61
	Hostility	8.21	5.78	0.00	21.00	0.71	−0.46
	Phobic Anxiety	9.64	7.55	0.00	26.00	0.55	−0.76
	Paranoid Ideation	10.29	5.44	0.00	23.00	0.29	−0.41
	Psychoticism	10.95	7.87	0.00	37.00	1.29	2.26
	Additional	11.13	5.87	2.00	22.00	0.02	−1.26
	Global Severity Index	3.44	1.71	0.11	3.44	0.02	−0.01
FSS‐III	Animal	22.13	11.38	0.00	50.00	0.26	−0.13
	Appraisal	29.87	12.48	4.00	51.00	−0.22	−1.00
	Social	17.78	9.50	0.00	36.00	0.12	−0.79
	Agoraphobia	10.28	5.87	0.00	23.00	0.23	−0.66
	Blood	15.89	8.00	3.00	31.00	0.35	−1.00
	Natural	17.09	7.84	3.00	32.00	−0.25	−0.69
	Total	156.84	64.26	18.00	288.00	−0.15	−0.37
SPIN	Fear	10.75	6.47	0.00	24.00	0.19	−0.78
	Avoidance	12.16	7.66	0.00	26.00	0.14	−1.06
	Physiological	6.84	4.15	0.00	16.00	0.29	−0.61
	Total Social Anxiety	29.75	17.68	0.00	63.00	0.14	−0.96

Abbreviations: ASI‐R, Anxiety Sensitivity Index—Revised; BDI‐II, Beck Depression Inventory—Second Edition; FSAQ, Four System Anxiety Questionnaire; FSS‐III, Fear Survey Schedule—Third Version; GAD‐7, 7‐item Generalized Anxiety Disorder; Kurt, kurtosis; *M*, mean; Max, maximum; Min, minimum; SCL‐90‐R, Symptom Checklist‐90‐Revised; SD, standard deviation; Skew, skewness; SPIN, Social Phobia Inventory.

As illustrated in Table 2, the clinical group obtained significantly higher mean total and composite scores than the nonclinical group. The skewness and kurtosis values for both groups were within acceptable ranges, indicating that the PDSS‐SR scores followed a relatively normal distribution in this sample. Notably, the clinical group's mean score was substantially higher than the recognized clinical threshold suggested in recent psychometric literature (e.g., Roberge et al. 2022).

Table 3 presents the descriptive profiles of the clinical group across various anxiety‐related measures. The mean scores on the BDI‐II and GAD‐7 reflect moderate to severe levels of depressive and generalized anxiety symptoms, aligning with typical comorbidity patterns observed in PD. While most subscales demonstrated acceptable distributional properties, some deviations in skewness and kurtosis were noted, particularly for the SCL‐90‐R Psychoticism subscale. These distributional characteristics further justify our use of nonparametric statistical methods to ensure the robustness of the subsequent convergent and discriminant validity analyses for the Persian PDSS‐SR.

The distributional properties of the data were evaluated through an examination of skewness and kurtosis. According to the criteria established by Kline (2023), the observed values for the majority of convergent measures, as well as in addition to the Persian PDSS‐SR total raw and composite scores, fell within the acceptable limits for assuming relative univariate normality. Furthermore, most of these indicators remained within the more stringent range of ±1, a threshold generally considered indicative of an essentially normal distribution suitable for psychological research (George and Mallery 2016). This finding was also supported by the guidelines provided by Gravetter and Wallnau (2017) regarding the adequacy of distributional shapes in behavioral sciences.

However, despite these indices of relative normality, we adopted a conservative methodological stance due to the modest size of the clinical group (*n* = 45). In smaller samples, standard errors can be relatively high, increasing the sensitivity of parametric tests to minor fluctuations. Therefore, to ensure maximum statistical robustness and to mitigate the risk of Type I errors arising from potential nonnormality or heteroscedasticity, Spearman's rank correlation (*r*_s_) was utilized for all subsequent validity analyses. This precautionary approach ensures that the results remain reliable and are not unduly influenced by minor distributional anomalies.

### 3.2 Validity

#### 3.2.1 Construct Validity

**FIGURE 1:**
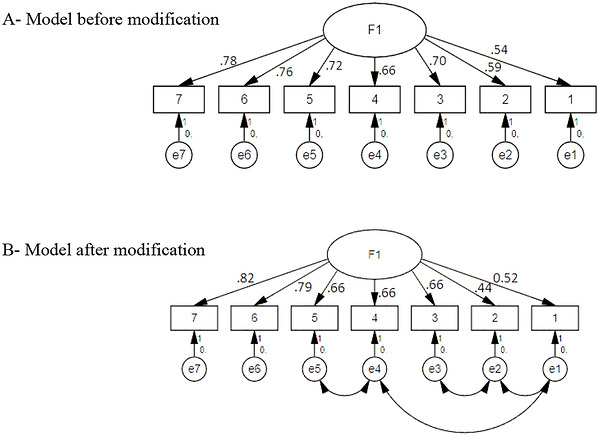
One‐factor structure of the PDSS‐SR—Persian Version (*n* = 45). (A) Model before modification. (B) Model after modification.

To evaluate the structural validity of the Persian PDSS‐SR, CFA was performed to test the established single‐factor model. Initial fit indices indicated a suboptimal model fit (e.g., RMSEA = 0.193, CFI = 0.816). To improve the model's performance, modification indices were examined, leading to the refinement of the model by allowing error terms between Items 1 and 2, Items 2 and 3, Items 4 and 5, and Items 1 and 4 to covary. These modifications were theoretically justified due to the semantic overlap between these items, which collectively address the frequency and immediate anticipation of panic symptoms (Items 1–3) and avoidance of situations and activities arising from attack anticipation (Items 4 and 5). Following these adjustments, the modified model achieved an excellent fit. Figure 1 displays the standardized factor loadings, while Table 4 summarizes the CFIs before and after the model modifications.

**TABLE 4:** Goodness of fit indices of Panic Disorder Severity Scale—Persian Version (*n* = 45).

Model	CMIN (*χ* ^2^)	df	CMIN/df	NFI	IFI	CFI	RMSEA
Before correction	37.036	14	2.645	0.747	0.826	0.816	0.193
After correction	11.091	10	1.109	0.924	0.992	0.991	0.050

Abbreviations: *χ* ^2^, chi‐square; CFI, comparative fit index; CMIN, minimum discrepancy; df, degree of freedom; IFI, incremental fit index; NFI, normed fit index; RMSEA, root mean square error of approximation.

As presented in Table 4, the final modified model demonstrated an excellent fit to the data. All fit indices (CFI, IFI, and NFI) and the absolute fit index (RMSEA) comfortably met the criteria for a robust model fit. Furthermore, all standardized factor loadings were statistically significant (*p* < 0.001), confirming the strong structural integrity of the single‐factor model in this clinical sample. These findings indicate that the Persian PDSS‐SR maintains the theoretical structure of the original scale.

#### 3.2.2 Convergent Validity

The correlation between the PDSS‐SR and the measures utilized for convergent validity was quite significant (*n* = 45, *p* < 0.05 and *p* < 0.01). As hypothesized, an increase in the total PDSS‐SR score was correlated with an increase in scores on most anxiety‐related subscales, indicating relatively strong convergent validity. Table 5 presents the Spearman's rank correlation coefficients (*r*_s_) that reflect this convergent validity.

**TABLE 5:** Convergent validity of the PDSS‐SR—Persian Version (*n* = 45).

Scale	Subscales/total scores	*r*_s_ with PDSS‐SR
ASI‐R	Cardiovascular–Digestive	0.25
	Respiratory	0.37^**^
	Fear of Observable Publicly Anxiety Reactions	0.37^**^
	Cognitive Dyscontrol	0.45^**^
BDI‐II	Total Depression	0.32^*^
FSAQ	Feeling	0.24
	Cognitive	0.10
	Behavioral	0.28
	Somatic	0.28
	Total	0.25
GAD‐7	Total Generalized Anxiety	0.49^**^
SCL‐90‐R	Somatization	0.43^**^
	Obsessive–Compulsion	0.33^*^
	Interpersonal Sensitivity	0.38^**^
	Depression	0.43^**^
	Anxiety	0.56^**^
	Hostility	0.06
	Phobic Anxiety	0.59^**^
	Paranoid Ideation	0.17
	Psychoticism	0.32^*^
	Additional	0.36^*^
	Global Severity Index	0.46^**^
FSS‐III	Animal	0.25
	Appraisal	0.45^**^
	Social	0.41^**^
	Agoraphobia	0.43^**^
	Blood	0.32^*^
	Natural	0.44^**^
	Total	0.47^**^
SPIN	Fear	0.31^*^
	Avoidance	0.35^*^
	Physiological	0.35^*^
	Total Social Anxiety	0.33^*^

Abbreviations: ASI‐R, Anxiety Sensitivity Index—Revised; BDI‐II, Beck Depression Inventory—Second Edition; FSAQ, Four System Anxiety Questionnaire; FSS‐III, Fear Survey Schedule—Third Version; GAD‐7, 7‐item Generalized Anxiety Disorder; SCL‐90‐R, Symptom Checklist‐90‐Revised; SPIN, Social Phobia Inventory.

* *p* < 0.05.

** *p* < 0.01.

As summarized in Table 5, the PDSS‐SR demonstrated significant positive correlations with the majority of the clinical instruments, supporting its convergent validity within the clinical sample. Specifically, the PDSS‐SR total score showed moderate to strong associations with generalized anxiety symptoms (GAD‐7). Regarding anxiety sensitivity, significant positive correlations were observed with most ASI‐R dimensions, with the exception of the Cardiovascular–Digestive subscale.

Furthermore, the PDSS‐SR was significantly linked to fear‐related symptoms (FSS‐III), social anxiety (SPIN), and the broader psychological distress indices of the SCL‐90‐R, particularly the Phobic Anxiety subscale and the GSI. Interestingly, under this robust nonparametric analysis, correlations with the FSAQ subscales did not reach statistical significance. Similarly, while a significant association was found with depressive symptoms (BDI‐II), the relationship with specific cognitive and feeling facets of the FSAQ remained nonsignificant. These findings collectively underscore the specific convergence of the PDSS‐SR with measures of anxiety and fear‐related pathology.

### 3.3 Sensitivity and Specificity

**FIGURE 2:**
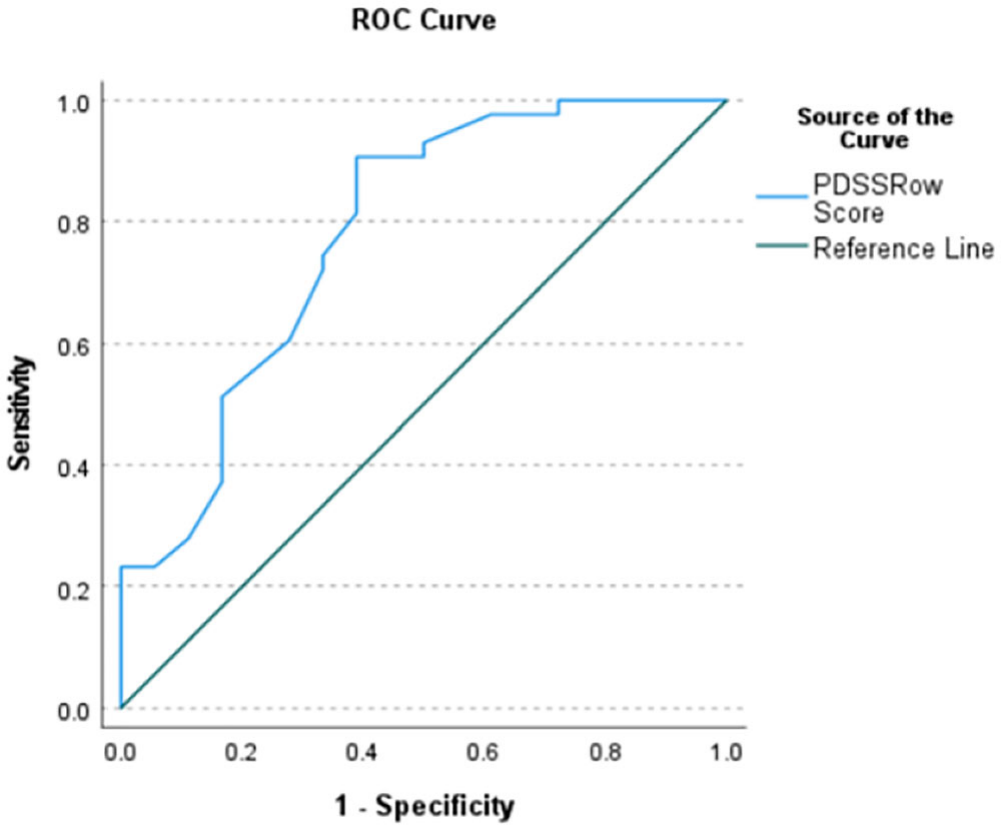
Receiver operating characteristic (ROC) curve of Panic Disorder Severity Scale—Persian Version (*n* = 63).

A receiver operating characteristic (ROC) plot constructed and analyzed based on the scores of participants with PD and those without (non‐PD) from the PDSS‐SR demonstrated an area under curve (AUC) that reflects an acceptable level of discrimination between the groups, according to Hosmer and Lemeshow (2000) and Manderkar (2010), as reflected in Figure 2. The optimal threshold, determined using the Youden index (Youden 1950), was found to be 6.5.

**FIGURE 3:**
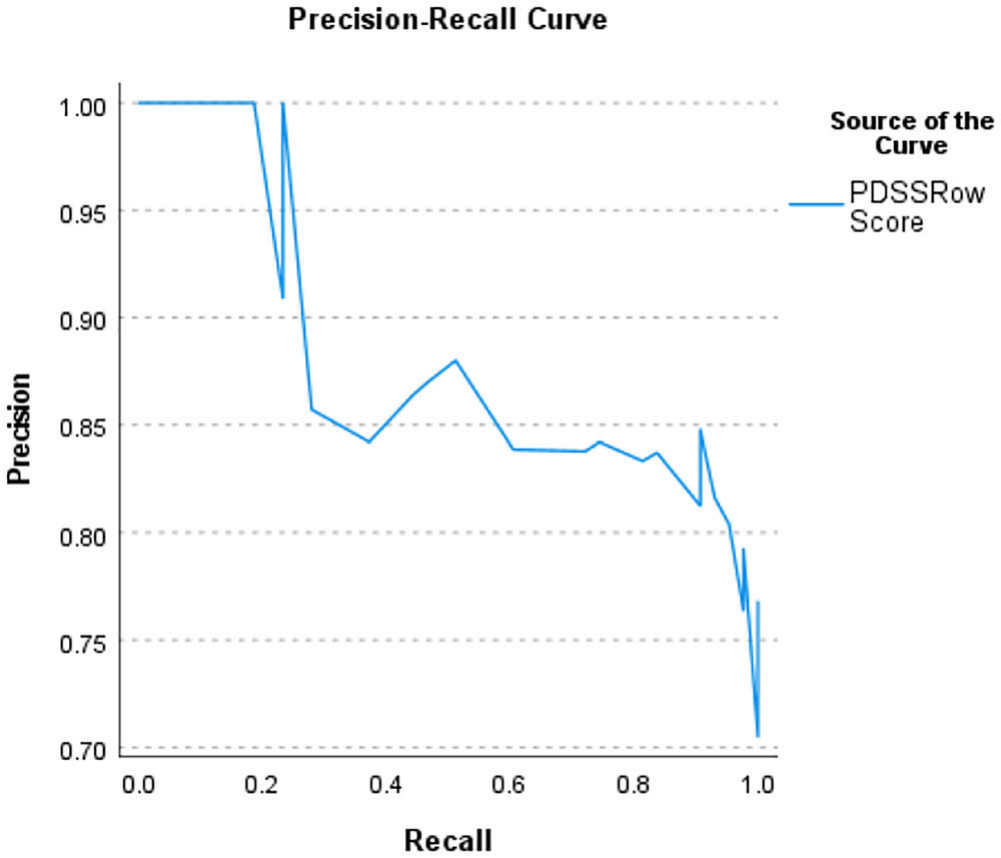
Accuracy–prediction diagram of PDSS‐SR—Persian Version (*n* = 63).

Although the likelihood ratios (LRs) suggest varying probabilities, the negative LR (LR−) indicates a high probability of accurately identifying negative cases (Edman and Runge 2014). The precision (positive predictive value [PPV]) and recall (sensitivity) further confirm that the majority of samples predicted to be positive for PD were indeed accurate based on the SCID‐5 diagnosis, as illustrated in the accuracy–prediction framework (Figure 3). Table 6 presents the area under the ROC curve, while Table 7 details the sensitivity, specificity, Youden index, Gini index, LR+ and LR−, and maximum Kolmogorov–Smirnov (K–S) statistic for the cut‐off point of 6.5 in the PDSS‐SR.

**TABLE 6:** Area under curve (AUC) of the receiver operating characteristic (ROC) curve (*n* = 63).

AUC	SE	95% CI	*p*
0.7804	0.06971	0.6437–0.9170	0.0006

Abbreviations: CI, confidence interval; SE, standard error.

**TABLE 7:** Operational characteristics of Panic Disorder Severity Scale—Persian Version (*n* = 63).

Cut‐off point according to the Youden index	> 6.500
Gini index	0.561
Youden index	0.518
Sensitivity % (CI)	90.70% (78.40%–96.32%)
Specificity % (CI)	61.11% (38.62%–79.69%)
LR+	2.332
LR−	0.152
Max K–S	0.518
Precision	0.848
Recall	0.907
*F* measure	0.877

Abbreviations: LR, likelihood ratio; Max K–S, maximum Kolmogorov‒Smirnov.

### 3.4 Reliability

The results demonstrated that the PDSS‐SR exhibited high internal consistency, surpassing the acceptable threshold of 0.85. Furthermore, Guttman split‐half reliability and test–retest reliability, assessed after a 6‐week interval using correlation and intraclass correlation coefficient (ICC), were also found to be satisfactory (see Tables 8 and 9).

**TABLE 8:** Reliability of PDSS—SR Persian Version (*n* = 45).

Coefficient tool	Cronbach's alpha	McDonald's omega	Guttman binomialization	Retest (*N* = 12; time interval: 6 weeks)			ICC
				Pearson	TAVI Kendall	Spearman	
PDSS‐SR	0.864	0.861	0.877	0.700^*^	0.571^*^	0.716^**^	0.855^**^

Abbreviation: ICC, intraclass coefficient.

* *p* < 0.05.

** *p* < 0.01.

**TABLE 9:** Item‐total statistics of Panic Disorder Severity Scale—Persian Version (*n* = 45).

Item	Scale mean if item deleted	Scale variance mean if item deleted	Corrected item − total correlation	Squared multiple correlation	Cronbach's alpha mean if item deleted
1	11.2543	27.553	0.505	0.496	0.850
2	10.9066	28.202	0.574	0.548	0.842
3	11.3680	26.384	0.635	0.569	0.832
4	11.6089	25.782	0.575	0.535	0.842
5	11.4086	24.272	0.685	0.530	0.824
6	11.2756	25.711	0.679	0.537	0.826
7	11.4311	24.576	0.693	0.606	0.823

The scale demonstrated an acceptable level of internal consistency, as evidenced by both McDonald's omega (McDonald 1999; McNeish 2018) and Cronbach's alpha (Cronbach 1951; 1957). Furthermore, the significant test–retest coefficient confirms the strong reliability of the PDSS‐SR, with an ICC that comfortably exceeds the recommended thresholds for clinical instruments (Koo and Li 2016). Detailed reliability coefficients are summarized in Table 8.

The data presented in Table 9 display the mean, variance, and Cronbach's alpha if any of the items are deleted. The overall Cronbach's alpha for the PDSS‐SR will decrease from the obtained total of 0.855 if any item is removed. Therefore, it does not seem logical to delete any of the items. This conclusion is further supported by the positive correlation between each item and the total score, excluding the item in question (corrected item − total correlation).

## 4 Discussion

This study aimed to determine the preliminary psychometric properties of the PDSS‐SR in Iranian samples with PD. First, the construct validity of the PDSS‐SR, assessed through CFA, yielded good to excellent fit indices (Hu and Bentler 1999), supporting a one‐factor structure, similar to the findings of Roberge et al. (2022). However, it should be noted that despite the original goal of the PDSS to measure the overall severity of panic attacks (Shear et al. 1997), different factor models on both one‐factor and two‐factor structures have been proposed in various cultures. The two‐factor model was confirmed based on the correlation of the first two items as the first factor and the subsequent five items as the second factor. The factor structure was not examined in the study of the self‐report version. Nevertheless, the one‐factor structure identified in the present study differs from the two‐factor structure reported by Svensson et al. (2018). These discrepancies may arise from differences in sample characteristics and cultural expressions of panic anxiety. Indeed, cross‐cultural research has emphasized that psychological instruments must demonstrate conceptual and structural equivalence across linguistic contexts, as cultural factors may influence response patterns and underlying factor structures if such equivalence is not empirically established (Dept 2024). Almost all of these two‐factor models exhibit unique loadings of the identified items on each factor. These two‐factor structures were derived from exploratory factor analysis or CFA conducted on samples ranging in size up to 5103 (Forsell et al. 2019). However, it is crucial to note that our recruitment process prioritized diagnostic stringency over sample breadth. Unlike studies utilizing convenient or community‐based samples, each participant in this study underwent a rigorous evaluation by both a clinical psychologist and a psychiatrist using the SCID‐5. By ensuring this gold‐standard diagnostic purity (Brodey et al. 2018), we maintained a highly homogenous clinical group, which is vital for psychometric accuracy in specialized populations. Furthermore, while larger samples are typically recommended for CFA, simulation studies suggest that acceptable parameter stability can be achieved in simple one‐factor models with small item numbers when factor loadings are adequate (Wolf et al. 2013). Given our clinical sample size (*n* = 45), the identified one‐factor model provides a parsimonious and stable framework for assessing panic severity in Iranian clinical settings, although further investigation with larger samples is warranted. Although for single‐factor models with a limited number of parameters, smaller samples may still yield stable and interpretable solutions, particularly in preliminary validation studies, the present findings should be interpreted with caution due to the relatively small clinical sample size.

Regarding convergent validity, the correlation of PDSS‐SR scores with scores on anxiety‐related scales—calculated using Spearman's rank correlation (*r*_s_) to account for the nonparametric distribution of the clinical data—ranging from 0.31 with the SPIN fear subscale to 0.59 with the SCL‐90‐R phobic anxiety subscale for significant correlation coefficients. Specifically, the PDSS‐SR showed moderate associations with generalized anxiety and phobic anxiety, while showing nonsignificant correlations with unrelated constructs such as hostility. These results align with the transdiagnostic nature of anxiety symptoms in the Iranian population, where panic symptoms often overlap with other anxiety sensitivities.

PD frequently co‐occurs with agoraphobia, and its core components—panic attacks, anticipatory anxiety, and avoidance—may function as partially independent but dynamically interacting processes. It seems that avoidance plays a central role in maintaining functional impairment and may be more strongly associated with overall clinical severity than panic frequency itself (Craske et al. 2021). Within the Iranian cultural context, where somatic expressions of psychological distress and heightened sensitivity to bodily sensations are relatively prominent, panic symptoms may be experienced and reported in ways that overlap with generalized anxiety and phobic responses. This overlap may reduce the specificity of associations between PDSS‐SR scores and certain anxiety subscales while strengthening others (e.g., phobic anxiety), thereby contributing to variability in correlation magnitudes. Accordingly, the observed pattern of associations likely reflects not only the latent structure of panic pathology but also its interaction with culturally shaped modes of symptom expression.

From a transdiagnostic perspective, these findings also suggest that the Persian PDSS‐SR captures a broader underlying vulnerability—likely rooted in anxiety sensitivity and emotional dysregulation—which transcends specific diagnostic boundaries. As mentioned, in the Iranian clinical population, this transdiagnostic core often manifests through somatization and autonomic overarousal, common to both panic and other emotional disorders. Therefore, the observed correlations with generalized and phobic anxiety subscales may reflect a shared psychopathological substrate rather than mere diagnostic overlap.

While most studies with larger samples reported stronger correlations (Roberge et al. 2022; Svensson et al. 2018), our findings demonstrate a consistent, albeit more conservative, pattern of convergence likely influenced by the modest sample size. Therefore, the hypothesis regarding the potential influence of sample size on the magnitude of these correlations appears to be tenable. Considering the findings related to construct validity and convergent validity—with the latter being confirmed through robust nonparametric estimates—the overall validity of the PDSS‐SR can be assessed as acceptable in a preliminary context, consistent with the reliability findings in this study.

In addition, in the present study, all measures were completed within a single assessment session following the SCID‐5 diagnostic interview; therefore, the data reflect a concurrent but temporally heterogeneous assessment context. Specifically, the PDSS‐SR evaluates panic symptoms over the past week, whereas several of the comparator instruments assess broader symptom experiences without a strictly defined short‐term reference period. Although all participants were instructed to respond based on their experiences during the past week, in accordance with the time frame of the PDSS‐SR, this temporal discrepancy may have introduced variability in the observed correlation patterns, as measures with differing time frames are likely to capture partially distinct aspects of anxiety symptomatology (state fluctuations vs. more stable tendencies). Consequently, the strength of associations between PDSS‐SR scores and other anxiety‐related constructs should be interpreted with consideration of these differences in temporal sensitivity. Accordingly, the findings primarily reflect a cross‐sectional snapshot of symptom severity within a narrowly defined assessment window rather than a longitudinal or trait‐level correspondence between constructs.

Since determining the cut‐off point strengthens the process of standardizing a diagnostic tool for broader clinical application, the present study also calculated the sensitivity and specificity of the raw score of the PDSS‐SR to achieve this goal. The sensitivity and specificity derived from the PDSS‐SR as a potential screening tool for PD identified an optimal cut‐off point of 6.50 according to the ROC curve, with a higher sensitivity of 90.70% and a specificity of 61.11%, based on the Youden index (Youden 1950). This profile highlights the strength of the PDSS‐SR as a screening tool, where high sensitivity is prioritized to ensure that clinical cases are not missed. However, using the Liu index (Liu 2012), a cut‐off point of 9.01 was established, which exhibited lower sensitivity (74.42%) but higher specificity (66.67%) compared to the Youden index, with an LR of 2.33. In French Canadian samples, Roberge et al. (2022) identified an optimal cut‐off point of 9, with 78.8% sensitivity, 70.4% specificity, and an AUC of 0.82. The cut‐off points obtained in the present study (6.50 based on the Youden index and 9.01 based on the Liu index) were relatively consistent with findings reported in the literature. The differing profiles of the curves may be attributed to the comparison group not suffering from PD. Furthermore, despite the limited sample size in this study, the findings regarding the sensitivity of the PDSS‐SR appear to be somewhat intermediate between those reported in Turkish (99%) and French Canadian (78.8%) samples, indicating reasonable consistency; however, this consistency does not extend to the specificity obtained (61.11% for the 6.500 cut‐off and 67.66% for the 9.01 cut‐off). It seems likely that a larger sample size would provide a more accurate comparison. Nevertheless, the specificity of 61.11% indicates that 61.11% of all individuals without PD would receive a negative result on the PDSS‐SR, highlighting the need to compare the false positive rate with the false negative rate. The slight variations in specificity may be attributed to clinical comorbidity patterns, such as the presence of agoraphobic avoidance, which was carefully monitored during the SCID‐5 diagnostic process.

The reliability of this instrument was assessed by calculating Cronbach's alpha, McDonald's omega, the Guttman split‐half coefficient, the test–retest correlation coefficient, and the ICC. The results from Cronbach's alpha (Cronbach 1957) and McDonald's omega (McDonald 1999), along with the test–retest correlation coefficient and ICC (Koo and Li 2016), indicated good levels of reliability, consistent with contemporary methodological standards (Kalkbrenner 2024), particularly given the limited clinical sample size. These findings align with the reliability indices reported in the PDSS‐SR research literature (Roberge et al. 2022). This reliability can be partly attributed to the culturally neutral nature of clinical symptomatology, although recent editions of the Diagnostic and Statistical Manual of Mental Disorders (DSM; i.e., DSM‐5 and DSM‐5‐TR) have underscored the significance of culture in defining mental disorders (APA 2022).

Taken together, these findings should be considered preliminary and interpreted within the context of sample size and potential comorbidity effects, highlighting the need for replication in larger and more diverse samples.

## 5 Limitations

In the present study, an effort was made to maintain a ratio of at least five subjects per item (Bentler and Chou 1987). Although this is the documented minimum ratio, it is essential to acknowledge the limited sample size (*n* = 45) as a primary limitation, which may affect the generalizability of the findings and the stability of the factor structure. To mitigate this, nonparametric statistical methods were employed to provide more robust estimates. In addition, the sampling method employed in this study resulted in a male‐to‐female ratio of approximately 1:4, which is approximately half of the overall ratio for PD as reported in the DSM‐5‐TR (1:2; APA 2022). This discrepancy represents another limitation, as it skews the findings toward women and restricts the generalizability of the results to men. Furthermore, while the current study focused on PD, the presence of comorbid conditions such as agoraphobia—although clinically monitored—remains a variable that should be more rigorously controlled in future large‐scale research. Achieving a gender ratio that is closer to that reported in the DSM's epidemiology of PD would facilitate a more accurate and reliable interpretation of the findings. Future studies with larger, multicenter samples are necessary to confirm these preliminary findings and further explore the cross‐cultural nuances of the Persian PDSS‐SR.

## 6 Research and Clinical Implications

In the present study, we aimed to assess the preliminary psychometric properties of the PDSS‐SR, despite the aforementioned limitations. A larger sample size would allow for a more comprehensive examination of construct validity through both CFA and exploratory factor analysis, thereby enabling a more precise exploration of discrepancies between studies.

In terms of clinical application, given the scale's high sensitivity observed in this Iranian sample, it may be considered a potentially useful screening tool, although the findings should be interpreted cautiously given the limited sample size. However, interviews are recommended to be conducted with clients during the administration of the PDSS‐SR to discuss the quality and severity of the symptoms reported in the instrument. This approach, combined with statistical and quantitative evaluations, allows for collecting more objective examples from clients' reports on the scale, leading to a more accurate diagnostic judgment regarding their conditions at the time of assessment. Furthermore, the transdiagnostic relevance of the PDSS‐SR in identifying panic‐related distress within the Iranian clinical population underscores its utility in busy psychiatric settings where a quick yet reliable assessment is needed.

## 7 Conclusion

This study provides evidence that the Persian PDSS‐SR possesses sufficient internal consistency, acceptable construct validity, and moderate convergent validity within the examined Iranian clinical sample. Using a conservative statistical approach (Spearman's rank correlation to account for the clinical distribution), the instrument showed a promising capacity to capture panic severity. An optimal cut‐off point of 6.50 was identified, offering high sensitivity for screening purposes. While these results are considered preliminary due to the modest sample size, the findings suggest that the Persian PDSS‐SR is a potentially useful and culturally adapted instrument for assessing PD severity in Iranian populations. Further large‐scale studies are warranted to confirm these initial psychometric observations.

## Author Contributions

**Fatemeh Akhavan‐Abiri**: conceptualization, investigation, writing – original draft, validation, visualization, writing – review and editing, software, data curation, formal analysis, project administration. **Mohammad Reza Shaeiri**: conceptualization, investigation, supervision, resources. **Hojjatollah Farahani**: methodology. **Arsia Taghva**: project administration, data curation, resources.

## Funding

This study was self‐funded and extracted from the PhD thesis on clinical psychology of the first author.

## Ethics Statement

The endorsement of the Research Ethics Committee of Shahed University (Tehran, Iran) has been obtained, bearing the identifier “IR SHAHED.REC.1402.126”.

## Consent

All participants provided written informed consent before participation. The study procedures were explained to all participants, and they were assured of the confidentiality and voluntary nature of their participation.

## Conflicts of Interest

The authors declare no conflicts of interest.

## Data Availability

The empirical data presented within this manuscript shall be made available upon reasonable request directed to the corresponding author in compliance with the principle of confidentiality.
